# ﻿On the species identity of a tropical oyster (Bivalvia, Ostreidae, *Dendostrea*) invading the eastern Mediterranean Sea

**DOI:** 10.3897/zookeys.1243.152856

**Published:** 2025-06-27

**Authors:** P. Graham Oliver, Matteo Garzia, Gustav Paulay, Daniele Salvi

**Affiliations:** 1 National Museum of Wales, Cathays Park, Cardiff CF10 3NP, UK National Museum of Wales Cardiff United Kingdom; 2 Department of Health, Life and Environmental Sciences, University of L’Aquila, Via Vetoio snc, 67100 L’Aquila-Coppito, Italy University of L’Aquila L’Aquila-Coppito Italy; 3 Florida Museum of Natural History, University of Florida, Gainesville, FL 32611, USA University of Florida Gainesville United States of America

**Keywords:** Alien species, bronze tree oyster, COI, DNA barcoding, leaf oyster, NIS, nomenclature, non-indigenous species, shell morphology, taxonomy, tree oyster, true oyster

## Abstract

Molecular and morphological data suggest that the Mediterranean populations of the non-indigenous genus *Dendostrea* are part of a single clade. This clade includes oysters from Rodrigues but is distinct from oysters from Hawaii and Mauritius. Based on morphology and sequence data, the Hawaiian and Mauritian oysters can be referred to as *Dendostreasandvichensis* Sowerby, 1871. The Mediterranean/Rodrigues clade, although morphologically very similar to *D.sandvichensis*, is significantly genetically distant from it and from *D.frons* and *D.folium*. As a result, the Mediterranean/Rodrigues clade cannot be assigned to any currently accepted nominal species. However, the statuses of the junior synonyms of *D.sandvichensis* are based on morphology and are therefore reconsidered with the result that *D.crenulifera* Sowerby, 1871 is shown to be morphologically very similar to the Mediterranean/Rodrigues clade. Given that the type locality of *D.crenulifera* is the Red Sea, and that Mediterranean populations are considered tropical invaders, *D.crenulifera* is a likely candidate name. However, without supporting sequence data from the type locality in the Red Sea, we conservatively conclude that the most appropriate name for the Mediterranean/Rodrigues clade is Dendostreacf.crenulifera (Sowerby, 1871).

## ﻿Introduction

Oysters identified as belonging to the genus *Dendostrea* were first recognised in the Mediterranean at the end of the 1990s (Çeviker 1999, 2001). Since then, the names *D.folium* (Linnaeus, 1758), *D.frons* (Linnaeus, 1758) and *D.crenulifera* (G.B. II Sowerby, 1871) have been applied, with the latter updated to *D.sandvichensis* (G.B. II Sowerby, 1871) following Huber (2010) and MolluscaBase eds (2025a). Crocetta et al. (2015) summarized records of non-indigenous oysters in the Mediterranean to that date, and recent papers (e.g., Ragkousis et al. 2023) record it as *D.folium* while others are undecided (e.g., Moussa and Khafage 2023). Currently, MolluscaBase eds (2021) recognises four other extant species: *D.cristata* (Born, 1778); *D.rosacea* (Deshayes, 1836), *D.trapezina* (Lamarck, 1819) and *D.senegalensis* (Gmelin, 1791). None of these names has ever been applied to Mediterranean *Dendostrea*.

MolluscaBase eds (2025b) currently gives a distribution map for *D.frons* indicating this species occurs in the Caribbean and the eastern Mediterranean, and thus suggests that the Caribbean Tree oyster is invasive in the eastern Mediterranean. There is a long history of confusion on the identities and type localities of *D.frons* and *D.folium*, but this was finally resolved by Dodge (1952) and accepted by Inaba and Torigoe (2004) and Huber (2010). *Dendostreafrons* is the accepted name for the “tree oyster” in the Caribbean while *D.folium*, the “leaf oyster”, is Indo-Pacific. The origin of Mediterranean *Dendostrea* from the Caribbean would appear to be atypical in that most eastern Mediterranean invasive species have a Lessepsian origin from the Red Sea via the Suez Canal (Albano et al. 2021a, b). However, given the discovery of *Dendostrea* shells attached to marine litter on the south-west coast of Ireland (Quigley and Hill 2015), a Caribbean origin cannot be totally excluded. The origins and potential nomenclature of the Mediterranean populations are important for the management of invasive species.

A molecular study of eastern Mediterranean *Dendostrea* populations (Delcour et al. 2025) revealed that all Mediterranean populations belonged to a single clade and that this clade was genetically distant from Caribbean *Dendostrea* identified as *D.frons* (Pagenkopp et al. 2015). Consequently, the identity of the Mediterranean populations remained obscure, and the purpose of this paper, using both molecular and morphological data, is to find the most appropriate nominal species identity. To identify the Mediterranean *Dendostrea* we begin with a molecular assessment based on DNA sequence data, and using morphology, ecology and biogeography, attempt to associate the resultant clades with recognised taxa. It must be stated at the outset that it has been very difficult to rely on the identification of *Dendostrea* species as applied in GenBank because, in most cases, names are attached to sequences based on shell features, sometimes combined with a geographic criterion (i.e., common species expected in that region). However, shell features are often misleading for oyster identification because of highly variable shell morphologies that overlap across species (Lam and Morton 2003; Wu et al. 2013; Salvi and Mariottini 2021; Salvi et al. 2021; McDougall et al. 2024). On the other hand, the geographic criterion becomes unreliable in the presence of sympatric morphologically similar species, and it is especially problematic with species identified as *D.frons* or *D.folium* due to their long-time confusion (Dodge 1952). Here we regard only sequences from the Caribbean as potentially belonging to *D.frons* and those from the Indo-Pacific as *D.folium*. At this time, all *Dendostrea* sequences are either not linked to any nominal taxa or only to *D.frons*, *D.folium* and *D.sandvichensis*. No sequences are available from *D.cristata*, *D.rosacea*, *D.trapezina* or *D.senegalensis*.

## ﻿Material and methods

### ﻿Material examined

**RODRIGUES** •17 specimens; Passage Grande Bassin; 19°39'S, 63°21.2'E; March 1999; attached to coral rubble, 1 m depth. PG Oliver leg.; NMW.Z.1999.073. • 7 specimens; Baie Nord; 19°42.7'S, 63°22.0'E; March 1999; attached to rocks at low tide; PG Oliver leg.; NMW.Z.1999.073. **MAURITIUS** • 18 specimens, Ile d’Ambre, Bassin Trou Polite, 20°2'S, 57°41.1E; March 1999; on intertidal rocks; PG Oliver leg.; NMW.Z.1999.073. **GREECE** • 1 specimen; Rhodes; 36°19.28'N, 28°12.62'E; May 2023; on intertidal rocks • 1 specimen; 36°26.59'N, 28°14.00'E; May 2023; on intertidal rocks • 2 specimens; 36°24.47'N, 28°13.78'E; May 2023; on intertidal rocks. **CYPRUS** • 1 specimen; 34°42.36'N, 33°15.45'E; May 2023; on intertidal rocks. • 1 specimen; 35°2.39'N, 32°24.03'E; May 2023; on intertidal rocks. • 1 specimen; 34°42.45'N, 33°10.18'E; May 2023, on intertidal rocks. • 1 specimen; 34°42.65'N, 33°10.33'E; May 2023; on intertidal rocks.

**Syntypes**: *Ostreacrenulifera* Sowerby, 1871, 2 shells, Red Sea. NHMUK 1879.2.26.242.

*Ostreasandvichensis* Sowerby, 1871, 2 shells, Sandwich Islands [= Hawaii] NHMUK 1900.2.13.28 and NHMUK 1912.6.4.4.

The specimens used here are a subset of the materials examined by Delcour et al. (2025) and only the specimens used in sequencing or imaged are listed here in Table 1.

**Table 1.** Specimens used in this study from the Mediterranean Sea (Med), Rodrigues (Rod), and Mauritius (Mau), along with geographical information and GenBank accession numbers.

Specimen ID	Voucher ID	Locality	Coordinates (Latitude and Longitude)	COI GenBank accession number
OS1391	OS1391	Greece: Rhodes	36°19.28'N, 28°12.62'E	PV453906
OS1403	OS1403	Greece: Rhodes	36°26.59'N, 28°14.00'E	PV453907
OS1412	OS1412	Greece: Rhodes	36°24.47'N, 28°13.78'E	PV453908
OS1415	OS1415	Greece: Rhodes	36°24.47'N, 28°13.78'E	PV453909
OS1490	OS1490	Cyprus	34°42.36'N, 33°15.45'E	PV453941
OS1522	OS1522	Cyprus	35°2.39'N, 32°24.03'E	PV453948
OS1503	OS1503	Cyprus	34°42.45'N, 33°10.18'E	PV453946
OS1480	OS1480	Cyprus	34°42.65'N, 33°10.33'E	PV453939
OS512	1999.073.005	Rodrigues, Passage Grande Bassin	19°39'S, 63°21.2'E	PV543794
OS516	1999.073.009	Rodrigues, Passage Grande Bassin	19°39'S, 63°21.2'E	PV543795
OS520	1999.073.013	Rodrigues, Passage Grande Bassin	19°39'S, 63°21.2'E	PV453888
OS533	1999.073.027	Rodrigues, Baie Nord	19°42.7'S, 63°22.0'E	PV543796
OS535	1999.073.029	Rodrigues, Baie Nord	19°42.7'S, 63°22.0'E	PV543797
OS536	1999.073.030	Rodrigues, Baie Nord	19°42.7'S, 63°22.0'E	PV453891
OS537	1999.073.031	Rodrigues, Baie Nord	19°42.7'S, 63°22.0'E	PV543798
OS538	1999.073.032	Rodrigues, Baie Nord	19°42.7'S, 63°22.0'E	PV453892
OS565	1999.073.060	Mauritius, Ile d’Ambre, Bassin Trou Polite	20°2'S, 57°41.1'E	PV453881
OS566	1999.073.061	Mauritius, Ile d’Ambre, Bassin Trou Polite	20°2'S, 57°41.1'E	PV453879
OS568	1999.073.063	Mauritius, Ile d’Ambre, Bassin Trou Polite	20°2'S, 57°41.1'E	PV453882
OS571	1999.073.066	Mauritius, Ile d’Ambre, Bassin Trou Polite	20°2'S, 57°41.1'E	PV543799
OS573	1999.073.068	Mauritius, Ile d’Ambre, Bassin Trou Polite	20°2'S, 57°41.1'E	PV453880
OS578	1999.073.073	Mauritius, Ile d’Ambre, Bassin Trou Polite	20°2'S, 57°41.1'E	PV453877
OS579	1999.073.074	Mauritius, Ile d’Ambre, Bassin Trou Polite	20°2'S, 57°41.1'E	PV453883
OS580	1999.073.075	Mauritius, Ile d’Ambre, Bassin Trou Polite	20°2'S, 57°41.1'E	PV453884
UF508018	UF508018	USA: Hawaii, Oahu, Kaneohe Bay, Kokokahi, fringing reef N of Likeke Place	21°24.89'N, 157°46.75'W	MW277847
UF508274	UF508274	USA: Hawaii, Oahu, Kaneohe Bay	21°30.0'N, 157°48.0'W	MW278296
UF508471	UF508471	USA: Hawaii, Oahu, Kaneohe Bay, Patch Reef 11	21°26.98'N, 157°47.77'W	MW278573
UF508473	UF508473	USA: Hawaii, Oahu, Kaneohe Bay, Patch Reef 11	21°26.98'N, 157°47.77'W	MW278575
UF508474	UF508474	USA: Hawaii, Oahu, Kaneohe Bay, Patch Reef 11	21°26.98'N, 157°47.77'W	MW278576
UF508476	UF508476	USA: Hawaii, Oahu, Kaneohe Bay, Patch Reef 11	21°26.98'N, 157°47.77'W	MW278577
UF508565	UF508565	USA: Hawaii, Oahu, Kaneohe Bay, Kaneohe Marine Corps Base, near airfield	21°27.12'N, 157°46.65'W	MW278695
UF508000	UF508000	USA: Hawaii, Oahu, Kaneohe Bay, Patch Reef 26	21°28.0'N, 157°49.08'W	MW284785

The material from Rodrigues and Mauritius was collected by PG Oliver during a Shoals of Capricorn field expedition in March 1999 and is deposited in the National Museum of Wales under NMW.Z.1999.073. Eight specimens from each island were selected for sequencing, while all 24 collected from Rodrigues and 18 from Mauritius were used in the morphological study. This material was identified as *Dendostreacrenulifera* Sowerby, 1871 (Oliver et al. 2004). The sequences of the Rodrigues and Mauritius specimens are deposited in GenBank, and their accession numbers are reported in Table 1.

Material from Hawaii was collected by G. Paulay; sequences are already deposited in GenBank (Table 1), and the voucher specimens are deposited in Florida Museum of Natural History.

The Mediterranean samples were collected by MG and DS and the vouchers are deposited in the Malacological collection of Department of MeSVA (University of L’Aquila). Eight specimens, four from Greece and four from Cyprus, were selected for sequencing. The GenBank accession numbers are reported in Table 1.

The type material examined is held by the Natural History Museum, London.

### ﻿Molecular assessment methods

For the molecular assessment, we used 24 oyster specimens identified as putative *Dendostrea* sp. from the Mediterranean Basin (*N* = 8), Mauritius (*N* = 8) and Rodrigues (*N* = 8) (Table 1) and we compared them with reference specimens of *D.sandvichensis*, collected from the type locality (Hawaii).

DNA extraction was performed from adductor muscle tissues fixed in ethanol 95% using the high-saline method (Sambrook et al. 1989). The mitochondrial COI barcode fragment was amplified through PCR using primers LCO1490 and HCO2198 (Folmer et al. 1994) using the protocol described in Salvi et al. (2010). Sanger sequencing of PCR products was carried out by the company Genewiz® (https://www.genewiz.com).

DNA sequences were processed and trimmed in Geneious Prime® v.2024.0.4 (Biomatters Ltd) and aligned with 51 sequences of *Dendostrea* mined from GenBank by taxon (“Dendostrea”[Organism]) and based on sequence identity > 95% with *Dendostrea* sequences obtained in this study (Table 1). This threshold is based on values of intra- and inter-specific distance estimated in previous studies (Liu et al. 2011; Salvi et al. 2014, 2022). Multiple sequence alignment was performed in MAFFT v.7.490 (Katoh 2002; Katoh and Standley 2013) using the G-INS-i algorithm with default parameters.

Phylogenetic assignment of Mediterranean *Dendostrea* oysters was performed in MEGA11 (Tamura et al. 2021) using the Neighbor-Joining (NJ) clustering method based on genetic distances computed under the Kimura-2-parameter (K2P) evolutionary model (Kimura 1980). Node support was assessed by the bootstrap method with 1000 replicates. The genus *Dendostrea* is not monophyletic in molecular phylogenies (Salvi et al. 2014; Salvi and Mariottini 2017, 2021; Li et al. 2021), therefore, this analysis aims to assign Mediterranean specimens to one of the *Dendostrea* clades rather than to provide a phylogeny of *Dendostrea*. The tree topology was visualized using FigTree v.1.4.4 (http://tree.bio.ed.ac.uk/software/figtree/) (Rambaut 2009). Genetic distances (*p*-distance and K2P distance) were calculated in MEGA 11.

### ﻿Morphological comparisons

Detailed morphological descriptions of *Dendostrea* species are few, making comparisons difficult. Stenzel (1971) and Torigoe (1981) noted the impact of the substrate on the morphology of *Dendostreafolium* and *D.frons* in which those attached to gorgonian stems are leaf-shaped and those attached to rocks are irregularly oval. Thus, the overall shape can be misleading but the size of the attachment area and the presence or absence of clasper spines can be informative. The external sculpture in the form (rounded or angled ribs) and number of ribs is cited by Torigoe (1981), although many shells can be totally overgrown or eroded. While the external surface is often obscured, the marginal plications give a good indication of the number of ribs. Internally, the presence, form and distribution of chomata are valuable characters, as is the internal colouration. In *Dendostrea*, lophine pustular chomata are typically present with or without marginal ostreine chomata. All of these characters were considered here.

Further complications arise when juvenile specimens are considered, as these may not show the adult characters as exemplified by Fig. 5G, which from sequence data is a juvenile *D.sandvichensis*. Consequently, only adult shells were considered in morphological comparisons.

## ﻿Results

### ﻿Molecular assessment

**Figure 1.**
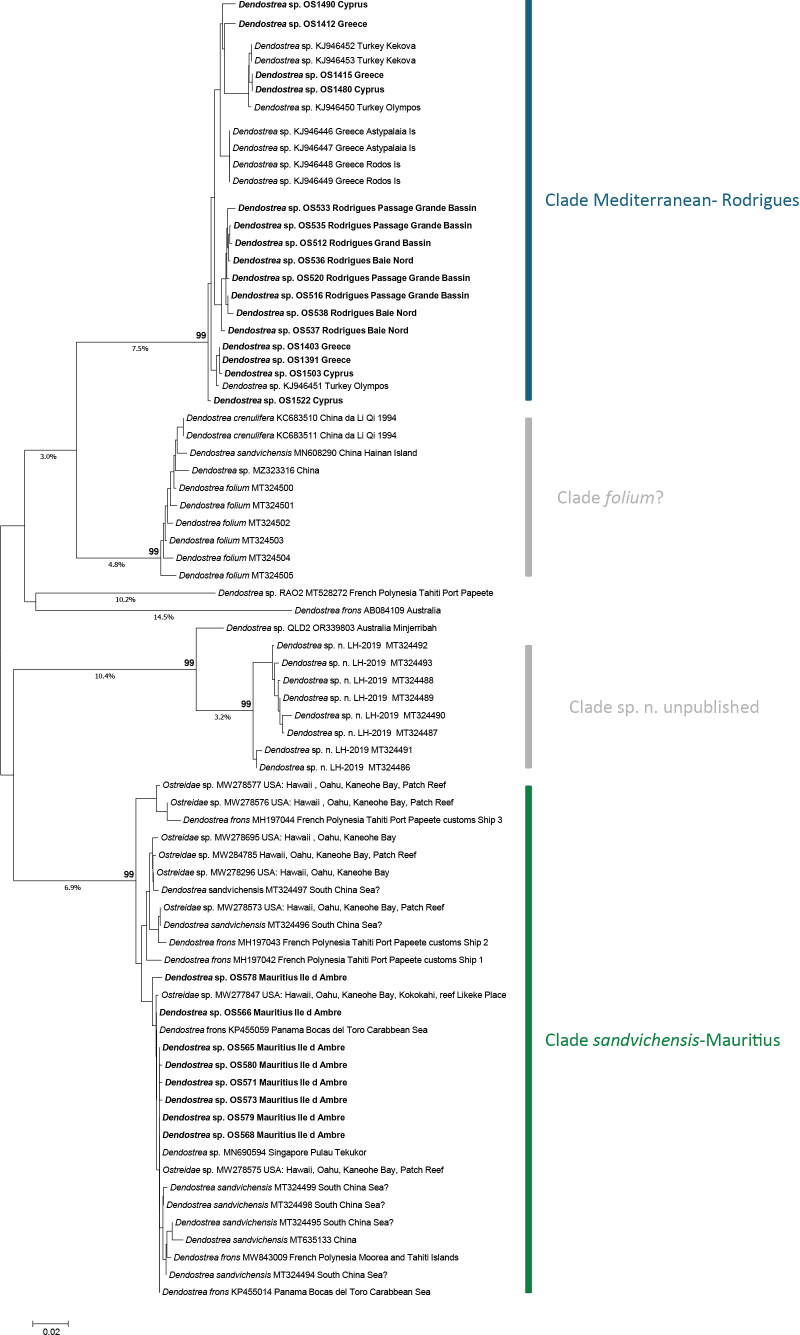
NJ tree of 75 COI sequences of *Dendostrea* based on Kimura-2-parameter genetic distance. Sequences generated in this study from Mediterranean, Rodrigues and Mauritius specimens are in bold. Bootstrap support is indicated above main nodes. Genetic distance values (> 3%) are reported below branches.

The NJ tree showed four main clades with high bootstrap support (BS = 99) and four lineages represented by a single sequence (Fig. 1).

All the Mediterranean *Dendostrea* sp. specimens clustered in a single clade that also included eight sequences of *Dendostrea* sp. from Turkey and Greece (Crocetta et al. 2015) as well as sequences from Rodrigues. Within this clade, genetic distances (*p*-distance) between Mediterranean and Rodrigues specimens ranged from 0.82 to 2.78% with a mean of 1.83% (Table 2).

**Table 2.** Mean genetic distance between specimens from the Mediterranean (Med), Rodrigues (Rod), Mauritius (Mau), and Hawaii (Haw). Values below the diagonal: Kimura-2-parameter (K2P) genetic distance; values above the diagonal: uncorrected (*p*-distance) genetic distance.

	Med	Rod	Mau	Haw
**Med**		1.83%	16.95%	16.85%
**Rod**	1.90%		16.91%	16.80%
**Mau**	22.88%	22.82%		1.55%
**Haw**	22.66%	22.60%	1.61%	

All *Dendostrea* sp. specimens from Mauritius clustered in a distinct clade together with topotypic specimens of *Dendostreasandvichensis* from Hawaii, plus other sequences from French Polynesia (from Ardura et al. 2021), China, and two sequences from Panama (Caribbean Sea; Pagenkopp et al. 2015). Within this clade genetic distances (*p*-distance) between Mauritius and Hawaii specimens ranged from 0 to 3.09% with a mean of 1.55% (Table 2).

As to the remaining two clades, one mostly includes unpublished sequences by Hu, Wang, and Guo (unpublished) of a new undescribed species from the South China Sea, labelled ‘*Dendostrea* sp. n. LH-2019’ in GenBank. The second clade includes, among others, six specimens identified as *D.folium* also from the South China Sea sequenced by Hu, Wang, and Guo (unpublished).

Mean genetic distance (*p*-distance) between Rodrigues and Mauritius specimens was 16.8% (K2P distance = 22.7%).

### ﻿Morphological assessment of Mediterranean and Mascarene *Dendostrea* oysters

#### ﻿Mediterranean *Dendostrea*

(Fig. 2)

Irregularly oval to lingulate shells rarely exceeding 5 cm maximum dimension. Lower valve with large attachment area, cupped, often asymmetric with the anterior side raised more than the posterior and externally sculptured with rounded radial ribs. Inner margin irregularly crenulate more so on the anterior edge. Upper valve weakly convex, often more or less flat, crenulations most developed around anterior ventral margin, external sculpture mostly poorly preserved remnants of radial folds at margins. Internally, both valves mostly lustrous white variously tinged green or pink and often with a reddish-brown margin. Simple chomata sparse on posterior dorsal margin elsewhere pustular (lophine) chomata present between crenulations and on posterior margin. Very occasional frondose morphs are present; here there is a narrow linear attachment reflected in the upper valve by a longitudinal median rib, radial ribs on both anterior and posterior.

**Figure 2.**
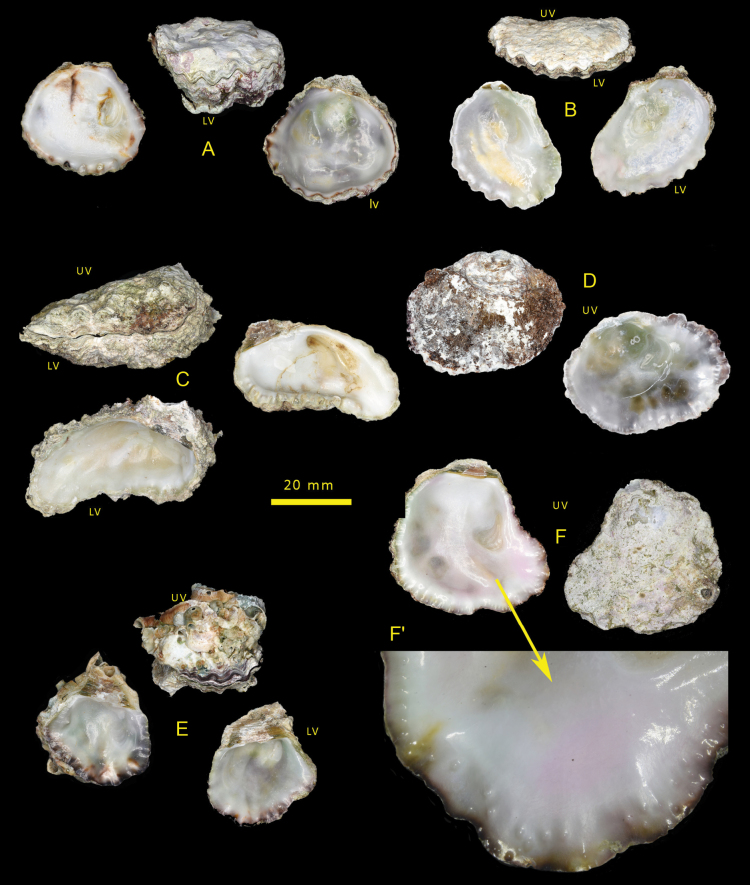
Dendostreacf.crenulifera shells from the Mediterranean **A** OS1412 Rhodes **B** OS1522 Cyprus **C** OS1415 Rhodes **D** OS1391 Rhodes **E** OS1490 Cyprus **F** OS1403 Rhodes.

#### ﻿Rodrigues *Dendostrea*

(Fig. 3)

Irregularly oval to lingulate shells rarely exceeding 4 cm maximum dimension. Lower valve with moderate to large attachment area, deeply to very shallowly cupped. Often asymmetric, the anterior side raised more than posterior and externally sculptured with rounded foliaceous radial ribs. Inner margin irregularly crenulate more so on the anterior edge, some undulating rather than crenulate. Upper valve weakly convex to almost flat, crenulations most developed around anterior ventral margin, external sculpture often obscure, low irregular foliaceous radial ribs in some. Inner margins irregularly crenulate more so on the anterior edge, some undulating rather than crenulate. Prominent simple chomata on lateral dorsal margins, pustular (lophine) chomata sparse or absent. Both valves internally white, some tinged with green, some with reddish-black margins. Very occasional frondose morphs are present; here there is a narrow linear attachment reflected in the upper valve by a longitudinal median rib, radial ribs on both anterior and posterior.

**Figure 3.**
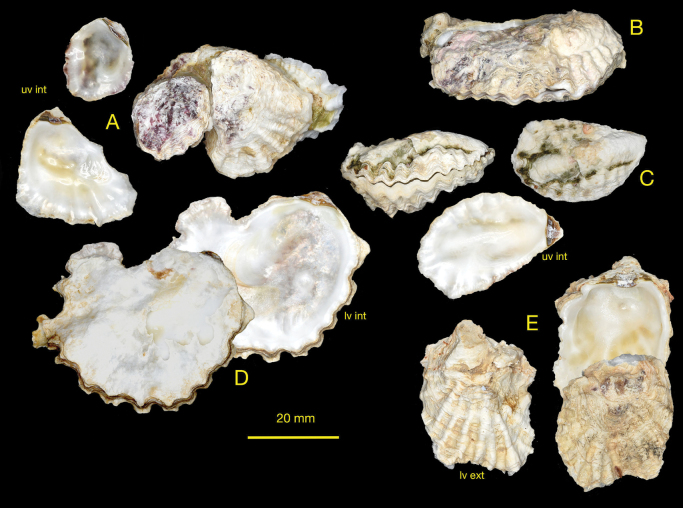
Dendostreacf.crenulifera shells from Rodrigues **A** NMW.Z.1999.073.30/31 **B** NMW.Z.1999.073.09 **C** attached to living coral, NMW.Z.1999.073.05 **D** NMW.Z.1999.073.29 **E** NMW.Z.1999.073.27.

#### ﻿Mauritius *Dendostrea*

(Fig. 4)

Irregularly oval to lingulate shells rarely exceeding 5 cm maximum dimension. Lower valve with moderate to large attachment area, cupped, often asymmetric the anterior side raised more than posterior and externally sculptured with rounded foliaceous radial ribs. Inner margin irregularly crenulate, more so on the anterior edge, some undulating rather than crenulate. Upper valve weakly convex, crenulations most developed around anterior ventral margin, external ribbing present in most, of low foliaceous radial or divergent ribs. Prominent simple chomata on all margins, pustular (lophine) chomata sparse or absent. Internally lower valve white, most tinged with some green, upper valve white, some with suffused purple black areas.

**Figure 4.**
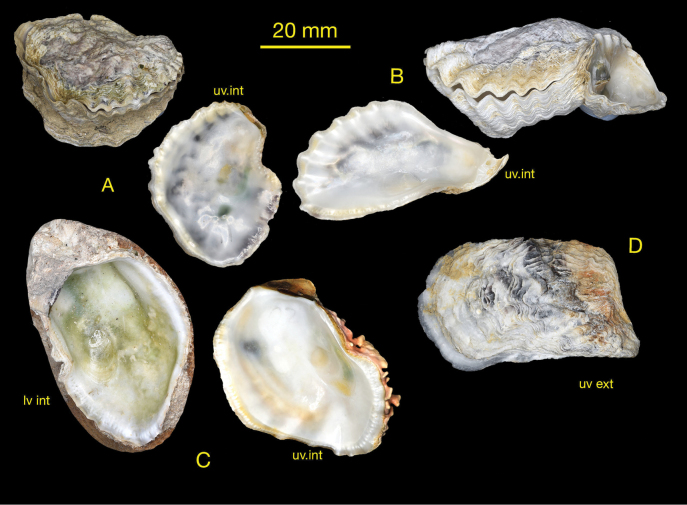
*Dendostreasandvichensis* shells from Mauritius. Ile d’Ambre, Mauritius **A** NMW.Z.1999.073.66 **B** NMW.Z.1999.073.63 **C** attached to living coral, NMW.Z.1999.073.74 **D** NMW.Z.1999.073.60.

#### ﻿Hawaii *Dendostrea*

(Fig. 5)

Irregularly oval to lingulate shells rarely exceeding 5 cm maximum dimension. Lower valve with moderate to large attachment area, cupped or very shallow, the latter externally sculptured with rounded foliaceous radial ribs. Inner margin irregularly crenulate, some undulating rather than crenulate. Upper valve flat to convex, crenulations matching lower valve, external ribbing present in most, of low foliaceous radial ribs. Prominent simple chomata on all margins, pustular (lophine) chomata most visible on ventral area. Internally lower and upper valves white, most tinged with some green or grey areas. External colouration mostly obscured but some show a pale shell with purplish to grey marks.

**Figure 5.**
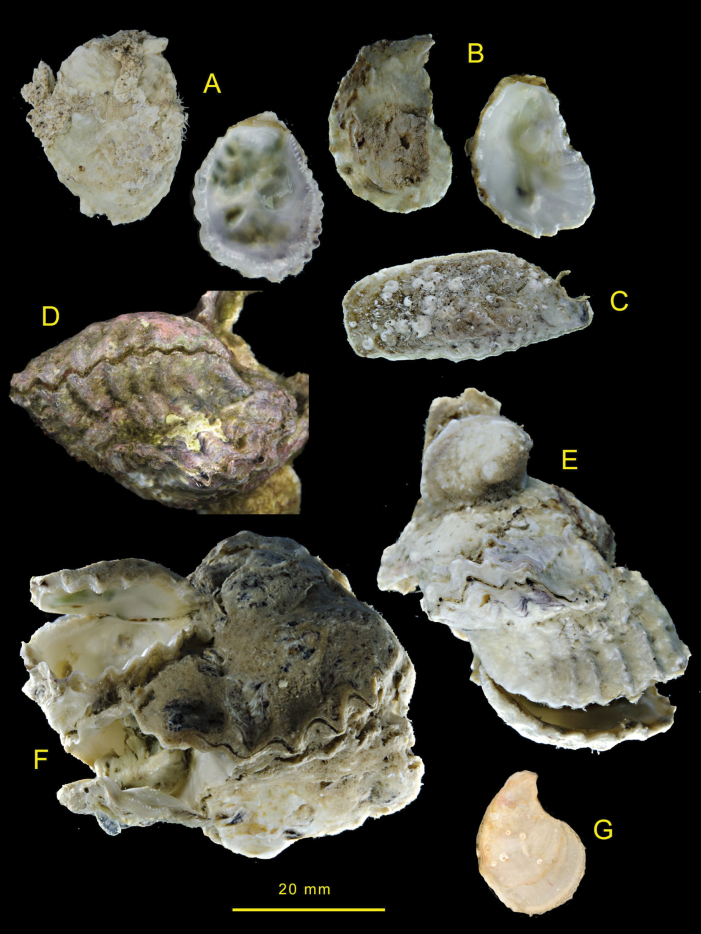
*Dendostreasandvichensis* shells from Hawaii. All Florida Museum of Natural History **A** UF508565 **B** UF508000 **C** UF544828 **D** UF511059 **E** UF511059 **F** UF510950 **G** UF508874, immature shells.

## ﻿Discussion

**Figure 6.**
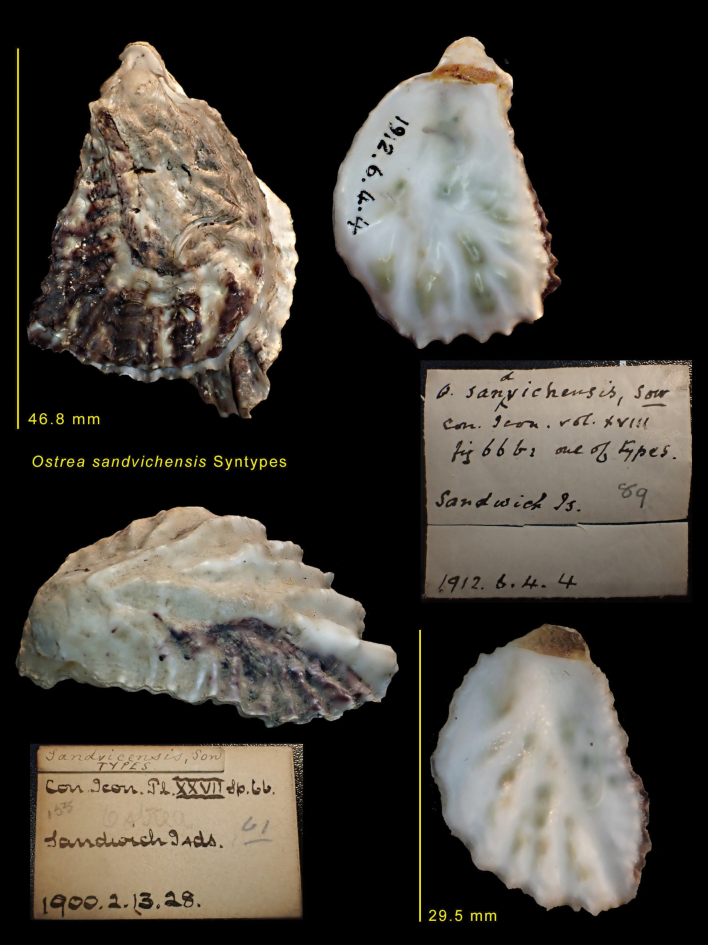
Syntypes of *Ostreasandvichensis* G.B. Sowerby II 1871. NHMUK 1879.2.26.242. Sandwich Islands (Hawaii).

Molecular data unambiguously identify four divergent clades that represent distinct species as the genetic distance (>20%) far exceeds the intraspecific values commonly observed within Ostreidae (<2.3%; Liu et al. 2011; Salvi et al. 2022). Although biogeographically disjunct, Mediterranean and Rodrigues oysters are conspecific and represent a distinct species from Mauritian oysters. The Mauritian sample clusters with material identified as *D.sandvichensis* taken from its type locality of Hawaii. Morphologically, they share the large attachment area, weak ribbing and internal colouration of white with darker markings seen in the syntypes of *O.sandvichensis* (NHMUK 1912.6.4.4 and 1900.2.13.28) (Fig. 6). The Mauritian sample can therefore be identified as *D.sandvichensis.* The inclusion of the specimens from Mauritius within the *D.sandvichensis* clade suggests that this taxon is widespread in the Indo-Pacific.

Less straightforward is the assignment of a species name to Mediterranean and Rodrigues oysters. Based on molecular data and morphological features, we can rule out that this species corresponds to either *D.frons* or *D.folium*. Caribbean material identified as *D.frons* clusters together with *D.sandvichensis*, casting doubt on their identification and at the same time confirming that there is no relationship between the Mediterranean *Dendostrea* and the Caribbean taxon. On the other hand, sequences from material identified as *D.folium* (‘Clade *folium*?’ in Fig. 1) suggest that this is a distinct taxon from both *D.sandvichensis* and the Mediterranean-Rodrigues species. From a morphological point of view, all the Mediterranean, Rodrigues and Mauritius shells are relatively small (< 50 mm in length) with a large attachment area, poorly developed ribbing and generally white internally. This is in contrast with Indo-Pacific *D.folium* which may reach 90 mm in length, has a small attachment area, and as the common name (Bronze tree oyster) infers, they are internally of a bronze hue. The upper valve ribbing is strong with deeply incised angulate ribs (see figures in Huber 2010:182; Inaba and Torigoe 2004: pl. 9 fig. 3; Oliver 1992: pl. 16 fig. 5a–c). We cannot confirm any sequences from specimens of this *D.folium* form. Although several specimens in one clade of the phylogenetic tree were identified as *D.folium* (‘Clade *folium*?’), unfortunately, no shell images or the proposed new species have ever been published. From the available sequence data and the shell morphology, it can be concluded that the Mediterranean population is not identifiable as *D.folium*.

Examination of shell morphological characters alone cannot discern between the Hawaiian, Mauritian, Rodrigues and Mediterranean samples given their remarkable similarity, with only subtle differences that are not consistent across all samples. The Mediterranean and Rodrigues shells are highly variable, but the expression of the external ribbing is poor in both, whereas the Mauritius shells are often distinctly ribbed, a feature seen in Hawaiian shells (Fig. 5). Some shells have a reddish-black margin in both populations but not in Mauritius. The crenulations are numerous and angulate in both, whereas the Mauritius and Hawaiian shells appear to have more rounded crenulations.

**Figure 7.**
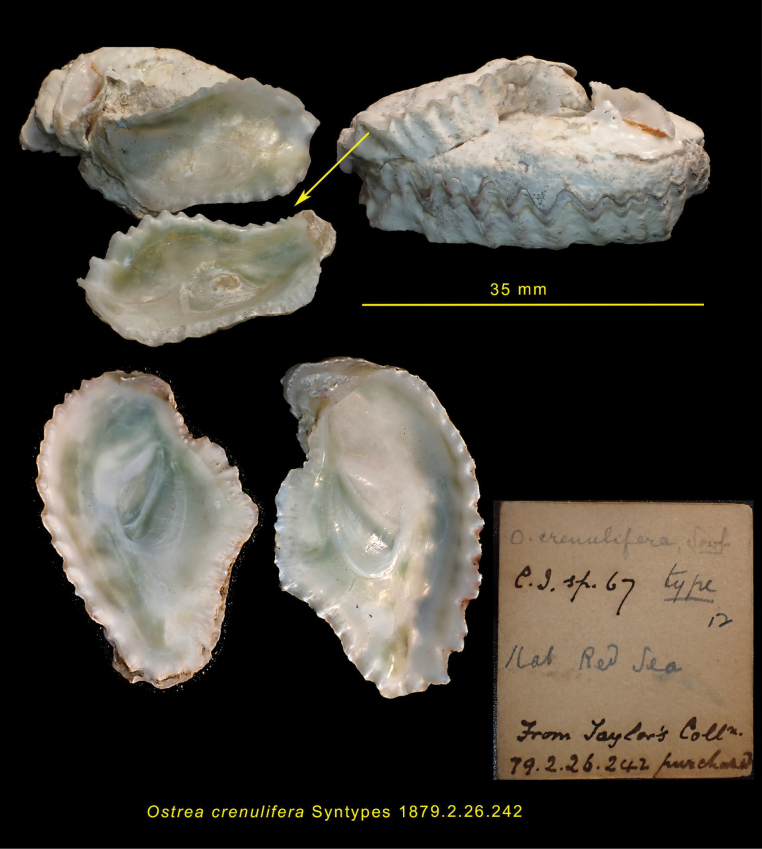
Syntypes of *Ostreacrenulifera* G.B. Sowerby II 1871. NHMUK 1900.2.13.28 and NHMUK 1912.6.4. Red Sea.

On the other hand, molecular data allow a straightforward diagnosis of *D.sandvichensis* from Hawaii and Mauritius, and the Mediterranean and Rodrigues samples, as these two species exhibit a large genetic divergence (Table 2). Mediterranean *Dendostrea* oysters are most likely to come from the Red Sea (Crocetta et al. 2015 and reference therein). *Dendostreasandvichensis* is recorded from the Red Sea but under the assumed synonym of *D.crenulifera* (Sharon et al. 2005; Galil 2007). Comparison with the type material of *Ostreacrenulifera* (NHMUK1874.2.26.2.2) (Fig. 7) reveals overall similarity in shell form and colouration, but at this time we have no sequences from Red Sea samples to confirm that the *crenulifera* form is distinct from *D.sandvichensis* or is part of the Mediterranean-Rodrigues clade. *Dendostreacrenulifera* was regarded as distinct by Lamy (1925) and was illustrated as distinct by Sharabati (1984, pl. 43, fig. 4a–d), Oliver (1992, pl. 16 fig. 6a–c), Bosch et al. (1995, fig. 994), Oliver et al. (2004, p. 3250) and Rushmore-Villeaume (2008, p. 188). A second assumed synonym of *D.sandvichensis* from the Red Sea is *Ostreaalabatra* Jousseaume in Lamy 1925, but that species was never illustrated and the type material has not been recognised.

However, given that we can show that the Mediterranean-Rodrigues clade is distinct from *D.sandvichensis*, an alternative name is required. On morphology alone, *D.crenulifera* seems appropriate, but without supporting molecular data such a decision may be premature, and the conclusion here is to use Dendostreacf.crenulifera (Sowerby, 1874) as the best fit.

This study confirms how the persistent challenge of reliably identifying oysters based on shell features results in a poor understanding of taxonomic identities, which are disconnected from GenBank sequence data. *Dendostrea* samples from the Mediterranean, Rodrigues, and Mauritius would certainly be considered conspecific based on shell features, and the close geographic proximity between Rodrigues and Mauritius further supports this interpretation. However, they represent two species with some of the greatest genetic divergence observed within the subfamily Ostreinae. At the same time, within any molecular clade, *Dendostrea* specimens with close genetic affinity have been classified as different species by various authors, rendering the GenBank database an unreliable reference for DNA barcoding applications.

## ﻿Additional information

### Author ORCIDs

P. Graham Oliver https://orcid.org/0000-0002-9481-0711

Matteo Garzia https://orcid.org/0000-0002-0918-9925

Gustav Paulay https://orcid.org/0000-0003-4118-9797

Daniele Salvi https://orcid.org/0000-0002-3804-2690

### Data availability

All of the data that support the findings of this study are available in the main text.
